# Severe Bilateral Sudden Sensorineural Hearing Loss Following Mild COVID‐19 Infection Requiring Cochlear Implantation: A Case Report

**DOI:** 10.1002/ccr3.72538

**Published:** 2026-04-15

**Authors:** Tena Šimunjak, Luka Županović, Filip Bacan, Andro Košec, Jakov Ajduk

**Affiliations:** ^1^ Department of Otorhinolaryngology, Head and Neck Surgery University Hospital Sveti Duh Zagreb Croatia; ^2^ Department of Otorhinolaryngology & Head and Neck Surgery University Hospital Center Sestre Milosrdnice Zagreb Croatia; ^3^ School of Medicine University of Zagreb Zagreb Croatia

**Keywords:** auditory rehabilitation, cochlear implantation, COVID‐19, sudden sensorineural hearing loss

## Abstract

Sudden sensorineural hearing loss (SSNHL) has been reported following COVID‐19, but profound irreversible hearing loss after mild infection remains rare. We describe a 28‐year‐old woman who developed severe bilateral SSNHL 3 weeks after confirmed mild COVID‐19, with complete deafness in the right ear and severe loss in the left ear. Hearing did not improve following systemic corticosteroids and hyperbaric oxygen therapy, and amplification provided minimal functional benefit. COVID‐19–related cochlear injury has been hypothesized to result from inflammatory and microvascular mechanisms, and post‐infectious cochlear fibrosis may complicate surgical rehabilitation. Despite these concerns, right‐sided cochlear implantation was successfully performed without evidence of cochlear fibrosis or ossification, leading to significant improvement in communication during auditory rehabilitation. This case highlights the potential for delayed, treatment‐refractory SSNHL even after mild COVID‐19 infection and supports cochlear implantation as an effective option in selected patients.

## Key Clinical Message

Severe sudden sensorineural hearing loss may occur as a delayed complication of mild COVID‐19 infection. Although infectious inner ear disease raises concern for cochlear fibrosis and may complicate implantation, cochlear implantation after COVID‐19–associated hearing loss is rare and can be successfully performed with favorable auditory outcomes.

## 1 Introduction

Sudden sensorineural hearing loss (SSNHL) is a challenging otologic condition characterized by a rapid decline in hearing, affecting one or both ears. The etiology of SSNHL is multifactorial and includes viral infections, vascular compromise, autoimmune disorders, and idiopathic causes. A recently published nationwide cohort study from South Korea involving over 6 million young adults demonstrated a significantly increased risk of hearing loss and SSNHL among individuals with COVID‐19 compared with those without infection [1].

Most published reports describe partial or complete hearing recovery following pharmacological treatment of COVID‐19–related SSNHL [2, 3]. However, considerable variability in clinical presentation, timing of onset, and disease severity has been observed. Consequently, the relationship between COVID‐19 infection and SSNHL remains an area of ongoing clinical and scientific interest. Herein, we report the case of a young, previously healthy patient who developed severe bilateral SSNHL as a delayed consequence of mild COVID‐19 infection, ultimately necessitating cochlear implantation. Written informed consent was obtained from the patient, and the study was prepared in accordance with the CARE case report guidelines.

## 2 Case History/Examination

A 28‐year‐old woman presented to the University Hospital Center Sestre Milosrdnice in Zagreb, Croatia, with sudden bilateral hearing loss occurring approximately 3 weeks after recovery from a confirmed mild COVID‐19 infection. The diagnosis of COVID‐19 was established by real‐time reverse transcription polymerase chain reaction testing of a nasopharyngeal swab. During the acute phase of infection, she reported mild dizziness lasting 2 days, without other systemic symptoms. Her past medical history was unremarkable.

**FIGURE 1:**
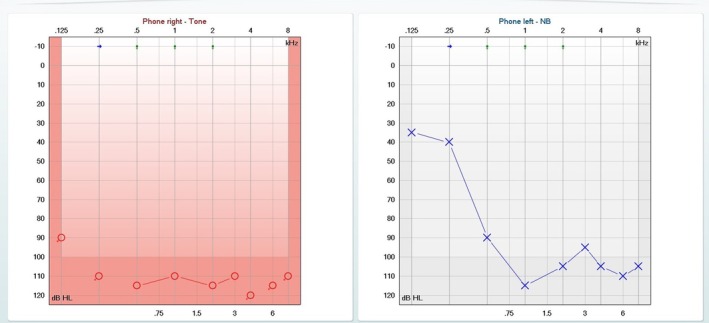
Pure‐tone audiometry prior to surgery demonstrating right‐sided deafness and severe sensorineural hearing loss in the left ear, with hearing thresholds ranging from 35 to 115 dB HL.

Pure‐tone audiometry demonstrated hearing thresholds ranging from 35 to 115 dB HL in the left ear, while the right ear showed complete deafness (Figure 1). Otoacoustic emissions were absent bilaterally. Otoscopic examination was unremarkable.

### 2.1 Differential Diagnosis, Investigations and Treatment

Magnetic resonance imaging and computed tomography of the temporal bones, as well as electroencephalography, revealed no pathological findings, effectively excluding retrocochlear, vascular, or structural causes of hearing loss. Serum testing showed elevated SARS‐CoV‐2 IgG antibody levels. The patient had not received COVID‐19 vaccination prior to infection. Initial treatment consisted of high‐dose systemic corticosteroid therapy (methylprednisolone 80 mg daily with gradual tapering), followed by 20 sessions of hyperbaric oxygen therapy. Despite these interventions, no hearing recovery was observed. A hearing aid trial in the left ear resulted in only minimal improvement in speech detection thresholds. Due to persistent right‐sided deafness and poor functional hearing, cochlear implantation was indicated.

### 2.2 Outcome and Follow‐Up

**FIGURE 2:**
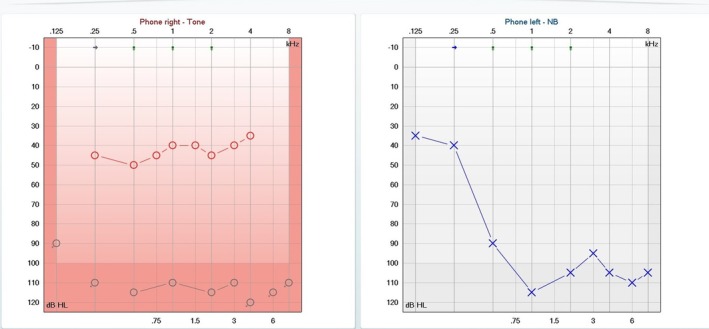
Postoperative pure‐tone audiometry demonstrating improved hearing thresholds in the right ear (35–50 dB HL) following cochlear implantation.

Cochlear implantation of the right ear was performed via a round window approach. Intraoperatively, no evidence of cochlear fibrosis or ossification was observed. Postoperatively, the patient demonstrated significant improvement in communication ability with the cochlear implant in the right ear combined with a hearing aid in the left ear during auditory rehabilitation (Figure 2).

## 3 Discussion

Since the emergence of the COVID‐19 pandemic, respiratory symptoms have represented the most frequently reported clinical manifestations. However, an increasing number of audiological and vestibular symptoms, including SSNHL, tinnitus, and dizziness, have been reported [2, 3]. Viral infections such as varicella‐zoster virus, cytomegalovirus, herpes simplex virus, Epstein–Barr virus, measles, mumps, adenovirus, and enteroviruses are well‐established causes of sensorineural hearing loss [4], and accumulating evidence suggests that SARS‐CoV‐2 may exert similar effects [1, 2, 3]. Unlike many viral infections in which SSNHL typically occurs during the acute phase, COVID‐19‐related SSNHL may present as a delayed manifestation, sometimes occurring weeks to months after mild infection in previously healthy individuals [2, 5]. Furthermore, SSNHL associated with COVID‐19 may be unilateral or bilateral and may occur in isolation or alongside tinnitus and/or dizziness, with each of these symptoms also reported independently in the setting of active SARS‐CoV‐2 infection without other systemic symptoms [6, 7]. Several studies have reported a significantly increased incidence of bilateral SSNHL among patients with COVID‐19 compared with the general population [2, 8]. Proposed mechanisms include virus‐induced inflammation, microvascular injury, and direct viral invasion of inner ear structures [4, 9]. Experimental studies have demonstrated expression of angiotensin‐converting enzyme 2, furin, and transmembrane protease serine 2 in cochlear hair cells and Schwann cells, facilitating SARS‐CoV‐2 entry and potentially compromising cochlear blood supply [10]. Additionally, cytokine‐mediated inflammatory responses may contribute to cochlear injury [3]. Ototoxic medications used in severe COVID‐19 cases have also been implicated [11, 12], although such agents were not administered in our patient. Most reported cases of COVID‐19‐associated audiovestibular symptoms demonstrate partial or complete recovery following medical therapy [2, 3]. In contrast, our patient exhibited progressive, treatment‐refractory hearing loss culminating in unilateral deafness. Only a limited number of cases describing deafness following COVID‐19 infection have been published, predominantly in patients with severe disease requiring intensive care [13, 14]. Cochlear fibrosis and ossification have been described following viral inner ear infections and may complicate electrode insertion [8]. In the context of COVID‐19, a case report has suggested that sensorineural hearing loss may, in some patients, be attributed to irreversible central involvement, potentially affecting the temporal lobe [15], which could further limit the effectiveness of cochlear impantation. In our patient, intraoperative findings revealed no fibrotic changes, enabling successful cochlear implantation. Although the long‐term efficacy of cochlear implantation in COVID‐19‐related deafness remains uncertain, the favorable outcome in this case supports its consideration in carefully selected patients.

## 4 Conclusion

The association between COVID‐19 and auditory manifestations remains incompletely understood, and standardized management guidelines are lacking. This case illustrates that severe, irreversible SSNHL may occur as a delayed complication of mild COVID‐19 infection, even in young and otherwise healthy individuals. When medical therapy fails, cochlear implantation may represent an effective rehabilitative option. Further research is required to elucidate the underlying mechanisms and optimize management strategies for COVID‐19‐associated hearing loss.

## Author Contributions

**Jakov Ajduk:** project administration, resources, supervision, validation, visualization, writing – review and editing. **Andro Košec:** resources, supervision, validation, visualization, writing – review and editing. **Filip Bacan:** conceptualization, validation, visualization. **Tena Šimunjak:** conceptualization, formal analysis, methodology, writing – original draft. **Luka Županović:** conceptualization, methodology, project administration, software.

## Funding

The authors have nothing to report.

## Consent

Written informed consent was obtained from the patient and the study was prepared according to the CARE case report guidelines.

## Conflicts of Interest

The authors declare no conflicts of interest.

## Data Availability

No data was collected in preparing the manuscript, and additional data regarding the patient is available from the corresponding author upon reasonable request.

## References

[1] H. J. Kim , S. Jeong , K. Kim , J. D. Lee , Y. H. Oh , and M. J. Suh , “Incidence of Hearing Loss Following COVID‐19 Among Young Adults in South Korea: A Nationwide Cohort Study,” EClinicalMedicine 75 (2024): 102759. 39175987 10.1016/j.eclinm.2024.102759 PMC11339059

[2] X. Meng , J. Wang , J. Sun , and K. Zhu , “COVID‐19 and Sudden Sensorineural Hearing Loss: A Systematic Review,” Frontiers in Neurology 13 (2022): 883749. 35572936 10.3389/fneur.2022.883749 PMC9096262

[3] Z. Jafari , B. E. Kolb , and M. H. Mohajerani , “Hearing Loss, Tinnitus, and Dizziness in COVID‐19: A Systematic Review and Meta‐Analysis,” Canadian Journal of Neurological Sciences 49, no. 2 (2022): 184–195. 10.1017/cjn.2021.63 PMC8267343 33843530

[4] B. E. Cohen , A. Durstenfeld , and P. C. Roehm , “Viral Causes of Hearing Loss: A Review for Hearing Health Professionals,” Trends in Hearing 18 (2014): 2331216514541361. 25080364 10.1177/2331216514541361 PMC4222184

[5] P. Viola , M. Ralli , D. Pisani , et al., “Tinnitus and Equilibrium Disorders in COVID‐19 Patients: Preliminary Results,” European Archives of Oto‐Rhino‐Laryngology 278, no. 10 (2021): 3725–3730. 33095432 10.1007/s00405-020-06440-7 PMC7582442

[6] K. M. McIntyre , N. M. Favre , C. C. Kuo , and M. M. Carr , “Systematic Review of Sensorineural Hearing Loss Associated With COVID‐19 Infection,” Cureus 13, no. 11 (2021): e19757. 34938632 10.7759/cureus.19757 PMC8684886

[7] E. Mehraeen , A. Afzalian , A. M. Afsahi , et al., “Hearing Loss and COVID‐19: An Umbrella Review,” European Archives of Oto‐Rhino‐Laryngology 280, no. 8 (2023): 3515–3528. 37093291 10.1007/s00405-023-07982-2 PMC10123565

[8] S. Shah , J. Rocke , K. France , and S. Izzat , “Sudden Sensorineural Hearing Loss in COVID‐19: A Case Series From the Wrightington, Wigan and Leigh Teaching Hospitals, United Kingdom,” Medical Journal of Malaysia 76 (2021): 55–59. 34558562

[9] R. F. Bento and T. V. Campos , “Hearing Loss, Tinnitus, and Dizziness and Their Relation With Covid‐19: What Is the Current Evidence?,” International Archives of Otorhinolaryngology 26, no. 1 (2022): e001–e002. 35096151 10.1055/s-0042-1742353 PMC8789497

[10] M. Jeong , K. E. Ocwieja , D. Han , et al., “Direct SARS‐CoV‐2 Infection of the Human Inner Ear May Underlie COVID‐19‐Associated Audiovestibular Dysfunction,” Communication & Medicine 1, no. 1 (2021): 44. 10.1038/s43856-021-00044-w PMC8633908 34870285

[11] V. Fancello , S. Hatzopoulos , V. Corazzi , et al., “SARS‐CoV‐2 (COVID‐19) and Audio‐Vestibular Disorders,” International Journal of Immunopathology and Pharmacology 35 (2021): 20587384211027373. 34142589 10.1177/20587384211027373 PMC8216371

[12] M. Dusan , S. Milan , and D. Nikola , “COVID‐19 Caused Hearing Loss,” European Archives of Oto‐Rhino‐Laryngology 279, no. 5 (2022): 2363–2372. 34235578 10.1007/s00405-021-06951-x PMC8263317

[13] K. Gerstacker , I. Speck , S. Riemann , A. Aschendorff , A. Knopf , and S. Arndt , “Deafness After COVID‐19?,” HNO 69, no. Suppl 2 (2021): 92–95. 34019138 10.1007/s00106-021-01041-0 PMC8138955

[14] C. Degen , T. Lenarz , and K. Willenborg , “Acute Profound Sensorineural Hearing Loss After COVID‐19 Pneumonia,” Mayo Clinic Proceedings 95, no. 8 (2020): 1801–1803. 32753155 10.1016/j.mayocp.2020.05.034 PMC7275185

[15] E. Cure and M. Cumhur Cure , “Comment on ‘Hearing Loss and COVID‐19: A Note’,” American Journal of Otolaryngology 41, no. 4 (2020): 102513. 32386897 10.1016/j.amjoto.2020.102513 PMC7192076

